# Creutzfeldt–Jakob disease in pregnancy: the use of modified RT-QuIC to determine infectivity in placental tissues

**DOI:** 10.1080/19336896.2021.1933872

**Published:** 2021-06-16

**Authors:** Collin C. Luk, Candace K. Mathiason, Christina D. Orrù, Gerard H. Jansen, Allison Thiele, Byron Caughey, Valerie L. Sim

**Affiliations:** a Division of Neurology, Department of Medicine, Faculty of Medicine & Dentistry, University of Alberta, Edmonton, Canada; b Department of Microbiology, Immunology, and Pathology, Prion Research Center, Colorado State University, Fort Collins, CO, USA; c Laboratory of Persistent Viral Diseases, Rocky Mountain Laboratories, National Institute of Allergy and Infectious Diseases, NIH, Hamilton, MT, USA; d Department of Pathology and Laboratory Medicine, University of Ottawa, Ottawa, Canada; e Department of Obstetrics and Gynaecology, Faculty of Medicine & Dentistry, Edmonton, Canada; f Centre for Prions & Protein Folding Diseases, University of Alberta, Edmonton, Canada

**Keywords:** Creutzfeldt–Jakob disease, pregnancy, Rt-QuIC, vertical transmission, prion

## Abstract

Sporadic Creutzfeldt–Jakob Disease (sCJD) rarely affects women of childbearing age. There is currently no evidence of vertical transmission. Given the biosafety implications of performing Caesarean sections (C-section) in these patients, we used sensitive real-time quaking-induced conversion (RT-QuIC) assays to test for the infectious prion protein (PrP^Sc^) in products of gestation. A 35-year-old woman with sCJD presented in her 10^th^ gestational week with an eight month history of progressive cognitive impairment. During C-section, amniotic fluid, cord blood and placental tissue were collected and analysed using RT-QuIC protocols adapted for use with these tissues. The patient’s diagnosis of sCJD, MM2 subtype, was confirmed at autopsy. There were borderline positive results in one sampled area of the placenta, but otherwise the cord blood and amniotic fluid were negative on our RT-QuIC assays. A healthy baby was delivered via C-section at 36 weeks and 3 days gestational age, with no evidence of neurological disease to date. We conclude that precautions should be taken with products of gestation, but the level of PrP^Sc^ is extremely low.

## Introduction

Creutzfeldt–Jakob disease (CJD) is a rapidly fatal, transmissible neurodegenerative disease that affects 0.3–1.7 people per million per year, peaking in the 7^th^ decade of life [1,2]. Rarely, CJD can affect women of childbearing age. There are seven previously reported cases of CJD (four sporadic, two iatrogenic, one variant) in pregnancy with no evidence of vertical transmission to date [3–9]. These studies primarily relied on immunohistochemistry screens of placental tissue, which were negative for infectious prion protein (PrP^Sc^). However, one study intracerebrally inoculated BALB/c mice with placental tissue and had subsequent evidence of transmitted disease [5]. The possibility that the placenta may contain low levels of PrP^Sc^ has implications for vertical transmission and risk management in delivery. Therefore, we applied the highly sensitive real-time quaking-induced conversion (RT-QuIC) assay to amniotic fluid, cord blood and placental tissue from a 35 year old pregnant woman with MM2 sporadic CJD (sCJD).

## Results

### PrP^Sc^ in gestational products

Placental tissue is not the usual testing material for RT-QuIC (usually done with CSF or brain homogenate). Therefore, several optimizations were performed in two separate labs, known for their expertise in developing RT-QuIC assays [10,11].

At the Caughey lab at the Rocky Mountain Laboratories (NIAID, NIH), known for the development and subsequent refinement of QuIC protocols, an iron oxide technique was used to concentrate samples. Control samples of placenta and cord blood were negative, but were able to support amplification if spiked with sCJD brain homogenate, even at 10^−7^ dilution. Three areas of placenta and cord blood were tested at dilutions of 10^−2^ to 10^−4^. Four replicates were completed with all results being negative. Amniotic fluid from our control and patient was also negative.

**Figure 1.**
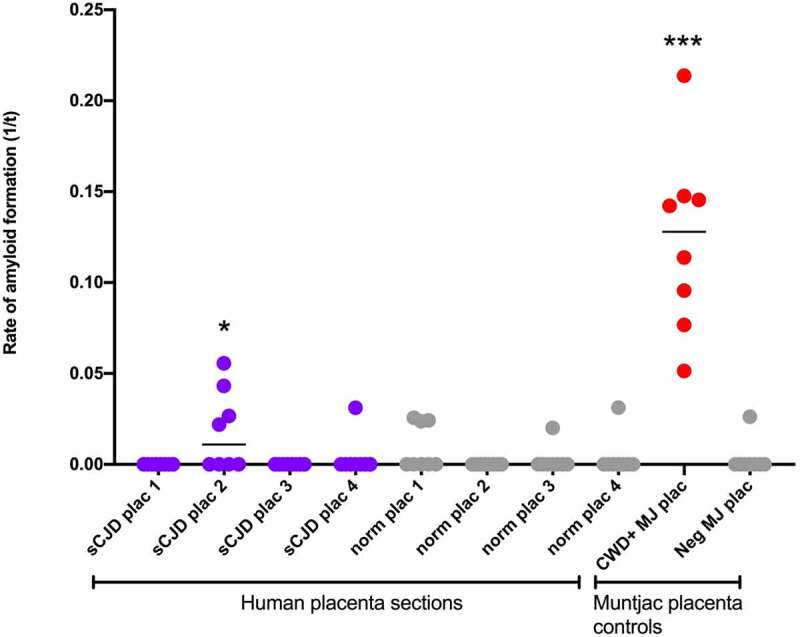
Placental homogenates taken from four unique sections of placenta with eight replicates each. There was significant positivity (p = 0.0153) in one section compared to control placenta (star). Positive control CWD+ Muntjac placenta and negative control Muntjac placenta were run concurrently with the assay. sCJD, sporadic Creutzfeldt–Jakob Disease; Norm, Normal control; Plac, placenta; CWD, chronic wasting disease; Neg, negative control; MJ, Muntjac

In the Mathiason lab (Colorado State), where RT-QuIC has been adapted for placental detection of prions in cervids infected with chronic wasting disease [10], four sections of placenta were tested with eight replicates each. One of the four placenta sections demonstrated significant positivity (p = 0.0153) (Figure 1). Amniotic fluid was subjected to iron oxide concentration and remained negative. Using an LIQ55 protocol on cord blood did not reveal any PrP^Sc^.

## Discussion

We present a case of sCJD in a pregnant patient with a healthy live birth. This is the eighth reported case of CJD during pregnancy and the fifth seen in sCJD [3–9]. While vertical transmission of CJD has been unequivocally demonstrated in animal, this has been less clear in human studies. Partially, this owes to the fact that CJD remains a rare disease and is even more rare with pregnancy. More importantly, as the incubation time for CJD can be prolonged, short-term follow-up with offspring of these patients may not reveal true transmission. Hence, studying PrP^Sc^ in products of gestation is important for understanding transmission risk in humans. This study is the first reported case to utilize the highly sensitive and specific diagnostic technique RT-QuIC and associated purification and enhancement techniques to detect PrP^Sc^ [12]. We did not find any evidence of PrP^Sc^ in amniotic fluid or umbilical cord blood. There was, however, a borderline result for one sampled area of placental tissue.

A previous pathological study of a pregnant sCJD patient was performed on amniotic fluid, cord blood, placenta and uterine wall tissue near the placental bed. These were all reported as being negative for PrP^Sc^. However, the study did not publish the protocol used for their prion detection [8]. In another study, the detection of PrP^Sc^ with western blot analysis was negative in amniotic fluid and uterine and placental homogenates. This study used enrichment protocols with immunoprecipitation by 6H4-conjugated beads and NaPTA [3].

Thus far, the greatest evidence for risk of vertical transmission was in a 38 year old woman with probable CJD (final autopsy not reported). The authors demonstrated that umbilical-cord leukocytes, placenta and breast milk (colostrum) were infectious at rates of 1/10, 5/8, and 2/10, respectively, when intracerebrally injected into BALB/c mice [5]. Interestingly, amniotic fluid was not infective. Importantly, inoculation of the patient’s blood plasma caused infections at a rate of 3/8. Whether infection from placental injection was due to contamination from blood plasma is difficult to exclude. Also, this case was presumed to be sCJD based on history, but genetic CJD cannot be excluded (no genotype presented) and the subtype of sCJD is not known. Given that different strains of prions can localize differently, it is important to consider which strain is analysed before concluding that all CJD cases will have a similar outcome [13]. Regardless, vertical transmission in humans has yet to be demonstrated, and with the level of PrP^Sc^ detection by RT-QuIC being so low and only in a minority of sampled areas, we suggest that exposure risks to those performing Caesarean sections on sCJD patients is extremely low.

## Patient and methods

### Clinical summary

A 35 year old woman in her tenth week of pregnancy was referred to the emergency department by the Reproductive Mental Health Programme with an 8-month history of cognitive decline. She had difficulty recalling names, spelling, and performing previously learned tasks. Her past medical history included learning disability, coeliac disease, polycystic ovarian syndrome, cholecystectomy and breast reduction surgery. She had one pregnancy 3 years prior with a normal delivery, but experienced non-progressive mood and memory changes after, which was thought to be due to post-partum depression. Her family history was non-contributory. Her family hunted and consumed wild deer, although she did not consume much venison personally. Her social history was non-contributory.

**Figure 2.**
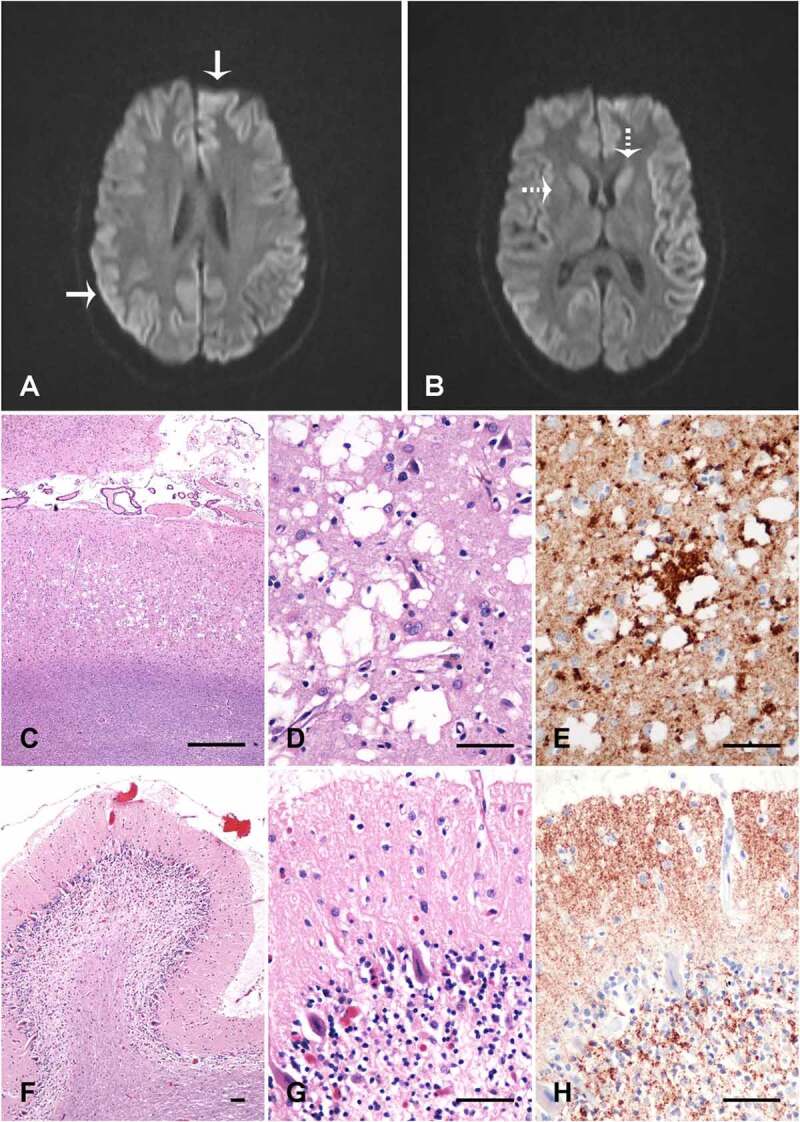
Neuroimaging and brain pathology. (A&B) Diffusion weighted imaging demonstrating cortical ribboning (solid arrow) and restricted diffusion in caudate and putamen (dotted arrow). Occipital neocortex (c-e) and cerebellum vermis (f-h) showing spongiosis with large coalescent vacuoles in the neocortex (c and d), and coarse perivacuolar PrP deposits (e), consistent with the MM2 phenotype of sporadic CJD. The cerebellum shows atrophy of the molecular layer, loss of internal granular layer cells (f and g), and synaptic PrP deposits in the molecular layer (h). C, D, F and G are haematoxylin and eosin stains; E and H are 12F10 prion protein immunolabelled (Cayman Chemical). The size bars are 500 µm (c) and 50 µm (d-h)

Her initial neurological exam demonstrated subtle limb dysmetria. Initial CSF studies were negative for autoimmune, inflammatory, infectious and neoplastic disease. Bloodwork showed a positive anti-nuclear antibody (ANA) and anti-double stranded-DNA, raising the possibility of systemic lupus erythematosus. Although full clinical criteria for lupus were not met, a trial of hydroxychloroquine was started. An electroencephalogram demonstrated bilateral diffuse slowing. A subsequent brain MRI showed restricted diffusion of the caudate and putamen with cortical ribboning (Figure 2(a,b)). A repeat lumbar puncture revealed elevated 14-3-3 (>80 000AU/mL) and Tau protein (>10,513 pg/mL) with positive EP-QuIC (endpoint quaking-induced conversion) assay, confirming the diagnosis of CJD. There was no *PRNP* gene mutation and she was homozygous M at codon 129.

Clinically, she rapidly worsened, with progressive ataxia, myoclonus, impaired cognition and aphasia. Following extensive discussions with the family and a multi-disciplinary team, expectant management was chosen, with maternal support to optimize neonatal outcomes. By 23 weeks gestational age, she developed sufficient aspiration risk that oral feeding could not provide adequate nutrition for the patient and pregnancy, so a nasogastric feeding tube was placed.

She was admitted to the antenatal ward for close monitoring and support at 28 weeks gestational age and remained there until delivery. Planned C-section at 36 weeks and 3 days gestational age was uncomplicated following amniocentesis, with the birth of a healthy female infant. After extubation, care goals were switched to palliation. She died 3.5 weeks after delivery. Autopsy confirmed the diagnosis of CJD consistent with the MM2 phenotype of sCJD (Figure 2(c-h)).

### Histology and pathology of brain tissue

Samples of brain tissue were taken from occipital neocortex and cerebellum. Haematoxylin and eosin stains were used in histology. Immunohistochemistry was performed with prion protein monoclonal antibody 12F10 (*Cayman Chemical*). All post-mortem tissue was analysed with consent from the family.

### Sample preparation

Amniotic fluid was collected through amniocentesis just prior to C-section. Cord blood and placenta were collected and stored in a −80°C freezer until evaluation. All samples were collected with consent from the family.

### Real-time quaking-induced conversion (RT-QuIC)

This technique exploits the ability of PrP^Sc^ to induce misfolding and aggregation of normal PrP *in vitro*. The beta sheet aggregates are detected by thioflavin T fluorescence [14]. Modifications to the assay allow it to be applied to different tissue types, including gestational tissues from animals [10,11]. All tissue and fluid analyses were performed post-mortem.

All reaction plates were run with four to eight technical replicates per sample using recombinant Syrian hamster PrP protein (amino acids 90–231). Controls included Alzheimer brain as negative control plus sCJD brain as positive control, or animal placenta from control or prion-infected (chronic wasting disease) Muntjac. Areas of placenta, amniotic fluid and cord blood were tested at dilutions of 10^−2^ to 10^−4^. Tissue controls were from a normal age-matched human gestation.

### Prion concentrating step

For some experiments, iron oxide nanoparticles were used to concentrate samples prior to RT-QuIC reactions without variation from our previously described protocol [15]. Briefly, iron oxide nanoparticle concentration was applied to 1 ml amniotic fluid with RT-QuIC analysis of undiluted, 1:10 and 1:100 dilutions. In other experiments, sodium phosphotungstate (NaPTA) precipitation was used as previously described [10]. Placental tissue (10 or 100 ml of a 10% homogenate) was subjected to NaPTA precipitation and assayed neat, 1:10 and 1:100. Amniotic fluid was also tested under conditions optimized for cerebrospinal fluid [16].

### Cord blood LIQ RT-QuIC

To further improve sensitivity, a lipase treatment step was added to the preparation of some cord blood samples. One millilitre of blood was lysed twice, washed with PBS, and re-suspended in 1 ml PBS before homogenizing the pellets. A LIQ55 procedure was performed on 100 µl of the resulting cellular homogenate as previously described [17], which includes lipase treatment, iron oxide bead extraction, and analysis by RT-QuIC at 55°C for 35 hours.

### RT-QuIC statistical analysis

Sample replicates were considered positive if they crossed the plate threshold (5 SD above the mean of the initial 5 readings). Amyloid formation rates for positive replicates were determined using the inverse of the time when each positive reaction exceeded a threshold value (1/time to threshold). Statistical analyses were run in GraphPad Prism. Mann–Whitney test was used to generate p-values by comparing the sample rates to the rates of known negative controls [10].
